# Spread of ceftriaxone non-susceptible pneumococci in South Korea: Long-term care facilities as a potential reservoir

**DOI:** 10.1371/journal.pone.0210520

**Published:** 2019-01-30

**Authors:** Min Joo Choi, Ji Yun Noh, Hee Jin Cheong, Woo Joo Kim, Min Ja Kim, Ye Seul Jang, Saem Na Lee, Eun Hwa Choi, Hoan Jong Lee, Joon Young Song

**Affiliations:** 1 Division of Infectious Disease, Department of Internal Medicine, Korea University College of Medicine, Seoul, Republic of Korea; 2 Department of Pediatrics, Seoul National University College of Medicine, Seoul, Republic of Korea; Carnegie Mellon University, UNITED STATES

## Abstract

Despite the availability of a pneumococcal National Immunization Program, which provides free PPSV23 vaccination for older adults aged ≥65 years in South Korea, pneumococcal pneumonia remains one of the most common respiratory infections, with increasing antimicrobial resistance. From January to December in 2015, all pneumococcal isolates were collected from a 1,050-bed teaching hospital in South Korea. All isolates were analyzed for serotype, genotype, and antimicrobial susceptibility. Demographic, clinical and microbiological data were compared between ceftriaxone susceptible and non-susceptible cases. Among 92 microbiologically identified pneumococcal isolates, ceftriaxone non-susceptible pneumococci (CNSP) accounted for 32 cases (34.8%). Some of these cases also showed levofloxacin resistance (25%, 8/32 isolates) and all CNSP cases were multidrug resistant. Compared to patients with ceftriaxone susceptible pneumococci (CSP), long-term care facility residents (odds ratio [OR] 7.0, 95% confidence interval [CI] 0.8–62.1) and patients with chronic lung (OR 4.1, 95% CI 1.1–15.0) and renal diseases (OR 9.1, 95% CI 1.2–70.5) were more common among those with CNSP on multivariate analysis. PPSV23-unique serotypes not included in PCV13 were more common in CNSP than in CSP (34.4% versus 13.3%, p = 0.02). Regarding genotypes, ST320 (10 cases), ST166 (7 cases) and ST8279 (3 cases) were dominant in CNSP, and ST8279 was only detected in previous long-term care facility residents. Clonal expansion and spread of CNSP strains should be monitored among patients with chronic lung/renal diseases and residents of long-term care facilities.

## Introduction

*Streptococcus pneumoniae* causes a broad spectrum of infectious diseases, ranging from mild upper respiratory illnesses to severe invasive diseases [1, 2]. Pneumonia and invasive pneumococcal disease (IPD) are the leading causes of morbidity and mortality after pneumococcal infection [2]. In South Korea, from 2011 to 2014, the estimated annual incidences per 100,000 persons were 44.3 and 4.8 cases for pneumococcal pneumonia and IPD, respectively [3]. The overall case-fatality rates were 9.1% and 30.8% for pneumococcal pneumonia and IPD, respectively, which increased with age [3]. The average per-capita medical fees for pneumococcal pneumonia and IPD were 1,887 and 7,562 USD, respectively [3–5].

Following its introduction in 1940, penicillin became the treatment of choice for pneumococcal diseases. However, there has been a change in regimen since the first penicillin-resistant pneumococci was found in Australia in 1967 [6]. Over the past 40 years, the resistance of pneumococci to penicillin and other antibiotics has been increasing dramatically worldwide, particularly in Asia [7–9]. Antimicrobial resistance rates in Asia are relatively high [7, 9]. In the Asian Network for Surveillance of Resistant Pathogens Study, which included patients with pneumococcal pneumonia in 11 Asian countries during 2008–2009, 59.3% of cases had multidrug-resistant (MDR) pneumococci. Korea has the third highest incidence of antimicrobial resistance (63.9%), following China and Vietnam [7]. Ceftriaxone non-susceptible pneumococci (CNSP) and levofloxacin non-susceptible pneumococci accounted for 3.2% and 5.2% of cases, respectively, which increased to 14.1% and 8.2% in subsequent research performed during 2012–2014 [9]. Despite increasing resistance rates, ceftriaxone is still recommended as the primary therapeutic regimen for pneumococcal diseases [10].

Considering the substantial disease burden of pneumococcal diseases and increasing resistance to various antibiotics, prevention through vaccination has become more important than in the past. Thus, pneumococcal vaccination was introduced as a national immunization program (NIP) in many countries, including the USA, Australia, and the UK [2, 11, 12]. In South Korea, pneumococcal vaccine is recommended for the elderly (age ≥65 years) and young adults (age 18–65 years) with comorbidities. Among them, pneumococcal polysaccharide vaccine (PPSV23) for the elderly has been included in the NIP since May 2013, and the vaccination rate has increased to 60% [13]. Moreover, in children, the 13-valent pneumococcal conjugate vaccine (PCV13) was introduced in March 2010 and included in the NIP in May 2014, when the vaccination rate had already reached 79.2% [14]. According to the US surveillance system, antimicrobial non-susceptible pneumococci among invasive diseases have reduced following vaccination [15]. Conversely, non-PCV13 serotype, MDR pneumococci (11A/E, 35B, 23A, 23B) have emerged [16–18]. In the era of PCV13, clinical and microbiological characteristics of MDR non-PCV13 serotype pneumococcal disease need to be further investigated.

This study primarily investigated clinicoepidemiological characteristics and serotype distribution of ceftriaxone non-susceptible pneumococcal diseases, and evaluated genetic diversity and antibiotic resistance profiles of ceftriaxone non-susceptible strains.

## Materials and methods

### Patients and pneumococcal isolates

All pneumococcal isolates regardless of age were collected from January to December 2015 at a 1,050-bed teaching hospital in Seoul, South Korea. For multiple isolates collected from a single infection episode, only the initial isolate was included in the study.

Demographic, clinical and laboratory data for each patient were collected from medical records and reviewed retrospectively. Demographic and clinical data included age, sex, body mass index, performance status, vaccination history, comorbidities, recent (≤90 days) healthcare exposure (recent hospitalization, surgery, antibiotic use, and long-term care facility residence), and clinical significance (true infection versus colonization). If the pneumococcal isolate was considered as the causative pathogen of infectious disease, we additionally gathered information regarding infection focus and 5-day clinical response. Pneumococcal vaccination status was determined by vaccination registration data in the National Immunization Registry Information System and medical records.

### Case definition

Ceftriaxone susceptible pneumococci (CSP) and CNSP were defined as isolates with ceftriaxone minimum inhibitory concentrations (MICs) of <2 μg/mL and ≥2 μg/mL, respectively. MDR was defined as non-susceptibility to three or more antimicrobial classes: β-lactam (penicillin or ceftriaxone), levofloxacin, erythromycin, linezolid, vancomycin, tetracycline, and trimethoprim-sulfamethoxazole. Extensive drug resistance (XDR) was defined as non-susceptibility to at least one agent in all antibacterial drug categories except vancomycin and linezolid. True infection was defined as any isolates from sterile fluid or respiratory isolates with clinical manifestation of pneumonia, which was diagnosed as follows: appearance of new radiological infiltrates and one or more relevant signs and symptoms: fever (temperature >38.2°C) or hypothermia (temperature <36.0°C); presence of purulent secretions; leukocytosis (>10,000 leukocytes/mm^3^) or leukopenia (<5,000 leukocytes/mm^3^). Colonization was defined as respiratory samples without clinical manifestations of pneumonia. Five-day treatment response was determined as improvement of all baseline symptoms, signs, and radiological features within 5 days. Performance status was evaluated using the Karnofsky Performance Status Scale [19].

### Serotyping, genotyping and susceptibility tests

The serotypes of all *S*. *pneumoniae* were determined with the capsular swelling (Quellung reaction) test using commercial pool and type/group-specific antisera (Statens Serum Institut, Copenhagen, Denmark) or the multiplex polymerase chain reaction assay recommended at www.cdc.gov/ncidod/biotech/strep/pcr.htm. MICs of ceftriaxone and other antimicrobial agents were determined using the broth microdilution method according to the guidelines of the Clinical and Laboratory Standard Institute (CLSI) [20]. *S*. *pneumoniae* ATCC 49619 was included as the quality control.

Multi-locus sequence typing (MLST) was performed according to a standard procedure [21]. The internal fragments of 7 housekeeping genes, i.e., aroE, gdh, gki, recP, spi, xpt, and ddl, were sequenced bi-directionally and identified by allele number and sequence type (ST) based on the pneumococcal MLST public database (http://spneumoniae.mlst.net). eBURST analysis grouped STs into clonal complexes (CCs) (Imperial College, London, UK) [22].

### Statistical analysis

We performed descriptive analyses and compared demographics and clinical characteristics between CSP and CNSP groups. To calculate ceftriaxone non-susceptibility and relate this to patient- and pathogen-specific factors, isolates were classified into two groups according to ceftriaxone susceptibility. The chi-square or Fisher’s exact test was used to compare the proportions of categorical variables between the two groups, and Student’s *t*-test was used to compare continuous variables between the groups. Clinically relevant factors and factors showing significant inter-group differences by univariate analysis were included in a multivariate logistic regression analysis. Data were analyzed using SPSS version 20.0 (SPSS Inc., Chicago, IL, USA). *P* values of <0.05 were considered statistically significant.

## Results

A total of 92 non-duplicate *S*. *pneumoniae* were isolated. The mean patient age was 66.1 (range 15–91) years and 72 (78.3%) patients were men (Table 1). In terms of their origins, seven (7.6%) isolates were from blood or other sterile fluid, while the remaining isolates (92.4%) were from respiratory samples. Among these, CNSP constituted 32 cases (34.8%). CNSP showed high levofloxacin resistance (25%, 8/32 cases) and MDR (100%, 32/32 cases) based on the 2015 CLSI breakpoints (Table 2). MIC_50_ and MIC_90_ values of antimicrobial agents were higher in the CNSP group than in the CSP group.

**Table 1:** Factors associated with ceftriaxone non-susceptible *Streptococcus pneumoniae* in adult patients.

	Total (N = 92)	Ceftriaxone susceptibility		Univariate	Multivariate analysis
		Susceptible (n = 60)	Non-susceptible (n = 32)	*p* value	OR (95% CI)
Age, years (range)	66.07 (15–91)	67.32 (15–91)	63.72 (18–88)	0.341	
Elderly aged ≥65 years, no. (%)	59 (64.1%)	38 (63.3%)	21 (65.6%)	0.827	2.587 (0.501–13.349)
Male, no. (%)	72 (78.3%)	49 (81.7%)	23 (71.9%)	0.278	
BMI <18, no. (%)	12/78 (15.4%)	7 (13.5%)	5 (19.2%)	0.521	
Performance status ≤70, no. (%)	29 (31.5%)	17 (28.3%)	12 (37.5%)	0.367	
Pneumococcal vaccination, no. (%)	30 (32.6%)	20 (35.7%)	10 (32.3%)	0.745	0.837 (0.229–3.060)
PPSV23, no. (%)	27 (29.3%)	18 (30.0%)	9 (28.1%)	0.851	
PCV13, no. (%)	7 (7.6%)	5 (8.3%)	2 (6.2%)	1.000	
Both, no. (%)	4 (4.3%)	3 (5.0%)	1 (3.1%)	1.000	
Nosocomial infection, no. (%)	13 (14.1%)	8 (13.3%)	5 (15.6%)	0.762	
Recent hospitalization, no. (%)	31 (33.7%)	20 (33.3%)	11 (34.4%)	0.920	
Recent surgery, no. (%)	7 (7.6%)	6 (10.0%)	1 (3.1%)	0.415	
Recent antibiotic use, no. (%)	18 (19.6%)	12 (20.0%)	6 (18.8%)	0.886	0.917 (0.246–3.415)
Recent cephalosporin use, no. (%)	13 (14.1%)	9 (15.0%)	4 (12.9%)	1.000	
Comorbid conditions, no. (%)	72 (79.3%)	47 (78.3%)	26 (81.2%)	0.742	
Smoking, no. (%)	45 (48.9%)	31 (58.5%)	14 (48.3%)	0.374	
Alcohol, no. (%)	17 (18.5%)	12 (21.4%)	5 (17.2%)	0.647	
Diabetes mellitus, no. (%)	28 (30.4%)	18 (30.0%)	10 (31.2%)	0.901	
Chronic heart disease, no. (%)	9 (9.8%)	5 (8.3%)	4 (12.5%)	0.714	
Chronic lung disease, no. (%)*	30 (32.6%)	17 (28.3%)	13 (40.6%)	0.231	4.116 (1.133–14.956)
Chronic liver disease, no. (%)	6 (6.5%)	4 (6.7%)	2 (6.2%)	1.000	
Solid cancer, no. (%)	23 (25%)	20 (33.3%)	3 (9.4%)	0.011	0.301 (0.042–2.143)
Chronic renal disease, no. (%)	8 (8.7%)	4 (6.7%)	4 (12.5%)	0.442	9.108 (1.177–70.458)
Immunosuppressive therapy, no. (%)	8 (8.7%)	8 (13.3%)	0	0.047	
Neuromuscular disease, no. (%)	27 (29.3%)	16 (26.7%)	11 (34.4%)	0.439	1.742 (0.458–6.630)
Pregnancy, no. (%)		0	0		
AACCI, mean (SD)	5.62	5.82 (2.884)	5.25 (3.121)	0.385	1.019 (0.722–1.438)
Long-term care facility residence, no. (%)	7 (7.6%)	2 (3.3%)	5 (15.6%)	0.047	7.049 (0.800–62.108)
Vaccine serotypes (PCV13+PPSV23), no. (%)	58 (63%)	31 (51.7%)	27 (84.4%)	0.002	9.219 (2.195–38.726)

OR, odds ratio; CI, confidence interval; BMI, body mass index; PPSV23, 23-valent pneumococcal polysaccharide vaccine; PCV13, 13-valent pneumococcal conjugate vaccine; AACCI, age-adjusted Charlson Comorbidity Index; SD, standard deviation

* In the ceftriaxone susceptible group, chronic lung disease referred to COPD (n = 13), bronchiectasis (n = 2), emphysema (n = 1), and interstitial lung disease (n = 1). In the ceftriaxone non-susceptible group, chronic lung disease referred to COPD (n = 7), asthma (n = 3), bronchiectasis (n = 2), and emphysema (n = 1).

**Table 2:** Antibiotic susceptibility to diverse antimicrobial agents.

	CSP (n = 60)				CNSP (n = 32)			
	MIC_50_	MIC_90_	Intermediate (%)	Resistant* (%)	MIC_50_	MIC_90_	Intermediate (%)	Resistant* (%)
PEN, meningitis	1	2	0 (0%)	43 (71.7%)	2/4	8	0 (0%)	32 (100%)
PEN, non-meningitis	1	2	4 (6.7%)	0 (0%)	2/4	8	10 (31.3%)	8 (25.0%)
CFT, meningitis	0.5	1	30 (50.0%)	1 (1.7%)	2/4	8	0 (0%)	32 (100%)
CFT, non-meningitis	1	1	0 (0%)	0 (0%)	2	8	16 (50.0%)	16 (50.0%)
Levofloxacin	0.5	1	0 (0%)	4 (6.7%)	0.5/1	16	0 (0%)	8 (25.0%)
Erythromycin	8	8	0 (0%)	41 (68.3%)	8	16	0 (0%)	32 (100%)
Linezolid	2	2	0 (0%)	0 (0%)	2	2	0 (0%)	0 (0%)
Vancomycin	0.5	0.5	0 (0%)	0 (0%)	0.5	0.5	0 (0%)	0 (0%)
Tetracycline	16	16	1 (1.7%)	39 (65.0%)	16	16	0 (0%)	31 (96.9%)
TMP/SMX	0.5/9.5	8/152	8 (13.3%)	17 (28.3%)	8/152	16/304	3 (9.4%)	29 (90.6%)
MDR, no. (%)	24/60 (40.0%)				32/32 (100%)			

MIC, minimum inhibitory concentration; CSP, ceftriaxone susceptible pneumococci; CNSP, ceftriaxone non-susceptible pneumococci; PEN, penicillin; CFT, ceftriaxone; TMP/SMX, trimethoprim/sulfamethoxazole; MDR, multidrug resistance

*The reference MIC break points used to define *in vitro* susceptibility and resistance were as follows: penicillin (meningitis), ≤0.06 mg/L and ≥0.12 mg/L; penicillin (non-meningitis), ≤2 mg/L and ≥8 mg/L; ceftriaxone (meningitis), ≤0.5 mg/L and ≥2 mg/L; ceftriaxone (non-meningitis), ≤1 mg/L and ≥4 mg/L; levofloxacin, ≤2 mg/L and ≥8 mg/L; erythromycin, ≤0.25 mg/L and ≥1 mg/L; linezolid, ≤2 mg/L (susceptible); vancomycin, ≤1 mg/L (susceptible); tetracycline, ≤1 mg/L and ≥4 mg/L; TMP/SMX, ≤0.5/9.5 mg/L and ≥4/76 mg/L.

### Factors associated with ceftriaxone non-susceptible *Streptococcus pneumoniae*

Overall, the pneumococcal vaccination rate was 32.6% (PPSV23, 29.3%; PCV13, 7.6%; both 4.3%), which was similar among both ceftriaxone-susceptible and non-susceptible cases (Table 1). Males were more dominant (78.3%), and comorbidities were accompanied in 79.3%, indistinguishable between the two groups. Compared to the CSP group, there was a higher proportion of long-term care facility residents (15.6% versus 3.3%, *p* = 0.047), and a lower proportion of solid cancers (9.4% versus 33.3%, *p* = 0.011) in the CNSP group, although neither finding reached statistical significance in multivariate analysis. In the multivariate analysis, chronic renal disease, chronic pulmonary disease, and vaccine serotype pneumococcal isolates were statistically significant risk factors for CNSP acquisition.

### Factors associated with 5-day treatment response among cases of true infection

Of 92 total isolates, 73 isolates were from true infection cases. Among these, four cases were excluded because treatment response could not be evaluated owing to follow-up loss (2 cases) and patient transfer (2 cases). Among 69 cases, 68 cases were pneumonia and one case was peritonitis; 48 (69.6%) of these cases showed clinical improvement within 5 days. Compared to non-responders within 5 days, responders were less severe in terms of qSOFA, CURB-65, and PSI score, although no scores reached statistical significance. Duration of antibiotic use and mortality rate were significantly higher in non-responders within 5 days (25.7 days versus 13.2 days, *p* = 0.003; 23.8% versus 4.2%, *p* = 0.024, respectively). In the univariate analysis, long-term care facility residents showed poor response within five days (*p* = 0.027), and duration of antibiotic use was significantly longer among poor responders (*p* = 0.003). In terms of antimicrobial susceptibility, penicillin resistance appeared to be associated with poor response (*p* = 0.065). On multivariate analysis, none of these relationships reached statistical significance, although elderly patients, long-term care facility residents, and those with nosocomial infections tended to show poor response within 5 days (Table 3).

**Table 3:** Factors associated with 5-day treatment response among cases with true pneumococcal infection.

	5-day response (n = 69)		Univariate	Multivariate analysis
	good (n = 48)	poor (n = 21)	*p* value	OR (95% CI)
Elderly aged ≥65 years, no. (%)	30 (62.5%)	17 (81.0%)	0.130	5.790 (0.752–44.562)
Male, no. (%)	40 (83.3%)	16 (76.2%)	0.515	
BMI <18 kg/m^2^, no. (%)	6 (14.3%)	3 (18.8%)	0.696	
Performance status ≤70, no. (%)	16 (33.3%)	10 (47.6%)	0.260	
Pneumococcal vaccination, no. (%)	17 (35.4%)	8 (38.1%)	0.831	1.073 (0.187–6.143)
PPSV23, no. (%)	17 (35.4%)	6 (28.6%)	0.579	
PCV13, no. (%)	0	5 (23.8%)	0.002	
Both, no. (%)	0	3 (14.3%)	0.025	
Nosocomial infection, no. (%)	4 (8.3%)	5 (23.8%)	0.119	6.644 (0.846–52.195)
Recent hospitalization, no. (%)	17 (35.4%)	6 (28.6%)	0.579	
Recent surgery, no. (%)	4 (8.3%)	0	0.306	
Recent antibiotic use, no. (%)	9 (18.8%)	5 (23.8%)	0.747	
Recent cephalosporin use, no. (%)	7 (14.6%)	3 (14.3%)	1.000	
Comorbid conditions, no. (%)	41 (85.4%)	15 (71.4%)	0.194	
Smoking, no. (%)	27 (61.4%)	10 (50.0%)	0.394	
Alcohol, no. (%)	10 (22.2%)	3 (14.3%)	0.526	
Diabetes mellitus, no. (%)	16 (33.3%)	6 (27.3%)	0.696	
Chronic heart disease, no. (%)	4 (8.3%)	3 (14.3%)	0.667	
Chronic lung disease, no. (%)*	17 (35.4%)	7 (33.3%)	0.867	0.457 (0.047–4.429)
Chronic liver disease, no. (%)	6 (12.5%)	0	0.167	
Solid cancer, no. (%)	11 (22.9%)	4 (19.0%)	1.000	
Chronic renal disease, no. (%)	4 (8.3%)	1 (4.8%)	1.000	
Immunosuppressive therapy, no. (%)	5 (10.4%)	1 (4.8%)	0.659	
Neuromuscular disease, no. (%)	14 (29.2%)	8 (38.1%)	0.464	4.234 (0.519–34.565)
AACCI, mean (SD)	5.50 (2.99)	5.67 (2.52)	0.824	1.023 (0.674–1.553)
LTCF residence, no. (%)	1 (2.1%)	4 (19.0%)	0.027	16.966 (0.722–398.656)
Pneumococcal serotype, no. (%)				
PCV13 serotype	22 (45.8%)	8 (38.1%)	0.551	
PPSV23 serotype	29 (60.4%)	13 (61.9%)	0.907	
PPSV23, not PCV13	10 (20.8%)	5 (23.8%)	0.761	
Antimicrobial susceptibility, no. (%)				
Penicillin resistance	2 (4.2%)	4 (19.0%)	0.065	2.425 (0.143–41.191)
Ceftriaxone resistance	8 (16.7%)	5 (23.8%)	0.515	
CNSP	18 (37.5%)	8 (38.1%)	0.963	
Erythromycin resistance	42 (87.5%)	16 (76.2%)	0.290	
Levofloxacin resistance	6 (12.5%)	5 (23.8%)	0.290	
MDR	33 (68.8%)	13 (61.9%)	0.579	
Admission, no. (%)	39 (81.2%)	17 (81.0%)	1.000	
Bacteremia, no. (%)	4 (11.1%)	3 (16.7%)	0.674	1.321 (0.045–39.159)
Concomitant viral infection, no. (%)	9/21 (42.9%)	5/13 (38.5%)	0.800	
Treatment, no. (%)				
Adequate empirical treatment	34 (73.9%)	13 (61.9%)	0.319	0.899 (0.142–5.703)
Ceftriaxone-based regimen	24 (50.0%)	8 (38.1%)	0.362	
Quinolone based regimen	19 (39.6%)	11 (52.4%)	0.324	
Severity parameters				
ICU admission, no. (%)	8 (16.7%)	6 (28.6%)	0.332	
Ventilator use, no. (%)	5 (10.4%)	5 (23.8%)	0.159	3.865 (0.182–81.991)
Septic shock, no. (%)	6 (12.5%)	5 (23.8%)	0.290	
qSOFA ≥2, no. (%)	10 (21.7%)	8 (40.0%)	0.126	1.767 (0.245–12.761)
CURB-65 ≥3, no. (%)	9 (20.5%)	8 (40.0%)	0.101	
PSI–IV or V, no. (%)	26 (56.5%)	14 (70.0%)	0.303	3.531 (0.287–43.474)
CRP (mg/L), mean (SD)	108 (99)	158 (136)	0.139	1.004 (0.996–1.012)
Procalcitonin (ng/mL), mean (SD)	3.2 (11.6)	5.7 (14.7)	0.453	
Lactic acid ≥2 mmol/L, no. (%)	10 (31.2%)	8 (44.4%)	0.351	
Cholesterol (mg/dL), mean (SD)	135 (46)	130 (52)	0.718	

OR, odds ratio; CI, confidence interval; SD, standard deviation; HTN, hypertension; BMI, body mass index; PPSV23, 23-valent pneumococcal polysaccharide vaccine; PCV13, 13-valent pneumococcal conjugate vaccine; AACCI, age-adjusted Charlson Comorbidity Index; LTCF, long-term care facility; CNSP, ceftriaxone non-susceptible pneumococci; MDR, multidrug resistance; qSOFA, quick Sequential Organ Failure Assessment score; PSI, Pneumonia Severity Index; SD, standard deviation

*Among 5-day good responders, chronic lung disease referred to COPD (n = 13), bronchiectasis (n = 2) and asthma (n = 2). Among 5-day poor responders, chronic lung disease referred to COPD (n = 4), emphysema (n = 1), interstitial lung disease (n = 1), and bronchiectasis (n = 1).

### Serotype distribution of *S*. *pneumoniae* isolates

All 92 isolates were serotyped. The major serotypes of *S*. *pneumoniae* were 19A (14.1%), 3 (13.0%), 11A (8.7%), 34 (7.6%), 35B (6.5%), 23A (5.4%), 10A (5.4%), and 19F (5.4%), accounting for 60.8% of all isolates (Table 4). Overall, the serotype coverage rates of PCV13 and PPSV23 were 42.4% and 59.8%, respectively. The serotype coverage rates of PCV13 and PPSV23 were somewhat higher in CNSP than in CSP. CNSPs accounted for 50% of PCV13 serotypes and 81.2% of PPSV23 serotypes, while CSP accounted for 38.3% of PCV13 serotypes and 48.3% of PPSV23 serotypes (Table 4). PPSV23 serotypes not included in PCV13 showed statistically significantly higher ceftriaxone non-susceptibility (34.4% versus 13.3%, *p* = 0.018). Specifically, serotypes 19A, 19F, and 11A were more prevalent in the CNSP group.

**Table 4:** Serotype distribution of *Streptococcus pneumoniae* isolates.

Serotype, no (%)		Total (N = 92)	Ceftriaxone susceptibility		*p* value
			Susceptible (n = 60)	Non-susceptible (n = 32)	
PCV13		39 (42.4%)	23 (38.3%)	16 (50.0%)	0.281
PPSV23		55 (59.8%)	29 (48.3%)	26 (81.2%)	0.002
PPSV23, not PCV13		19 (20.7%)	8 (13.3%)	11 (34.4%)	0.018
PCV13	19F	5 (5.4%)	1 (1.7%)	4 (12.5%)	
	23F	1 (1.1%)		1 (3.1%)	
	6B	2 (2.2%)	2 (3.3%)		
	9V	2 (2.2%)	1 (1.7%)	1 (3.1%)	
	18C	1 (1.1%)	1 (1.7%)		
	19A	13 (14.1%)	4 (6.7%)	9 (28.1%)	
	3	12 (13.0%)	12 (20%)		
	6A	3 (3.3%)	2 (3.3%)	1 (3.1%)	
PPSV23, not PCV13	9N	2 (2.2%)	1(1.7%)	1 (3.1%)	
	10A	5 (5.4%)	5 (8.3%)		
	11A	8 (8.7%)	1 (1.7%)	7 (21.9%)	
	15B	1 (1.1%)		1 (3.1%)	
	17F	1 (1.1%)	1 (1.7%)		
	20	2 (2.2%)		2 (6.3%)	
Others	23A	5 (5.4%)	3 (5.0%)	2 (6.3%)	
	34	7 (7.6%)	7 (11.7%)		
	35B	6 (6.5%)	6 (10.0%)		
	6C	2 (2.2%)	2 (3.3%)		
	6D	1 (1.1%)	1 (1.7%)		
	7B/C	1 (1.1%)	1 (1.7%)		
	11F/B/C	1 (1.1%)	1 (1.7%)		
	13	2 (2.2%)	2 (3.3%)		
	15A	2 (2.2%)	1 (1.7%)	1 (3.1%)	
	16Aor36	1(1.1%)	1 (1.7%)		
	16F	1(1.1%)	1 (1.7%)		
	17A	1(1.1%)	1 (1.7%)		
	31/40	1 (1.1%)		1 (3.1%)	
	27/32/41	1 (1.1%)	1 (1.7%)		
NT		2 (2.2%)	1 (1.7%)	1 (3.1%)	

PCV13, 13-valent pneumococcal conjugate vaccine; PPSV23, 23-valent pneumococcal polysaccharide vaccine; NT, non-typeable

### Distribution of sequence types and serotypes

**Fig 1:**
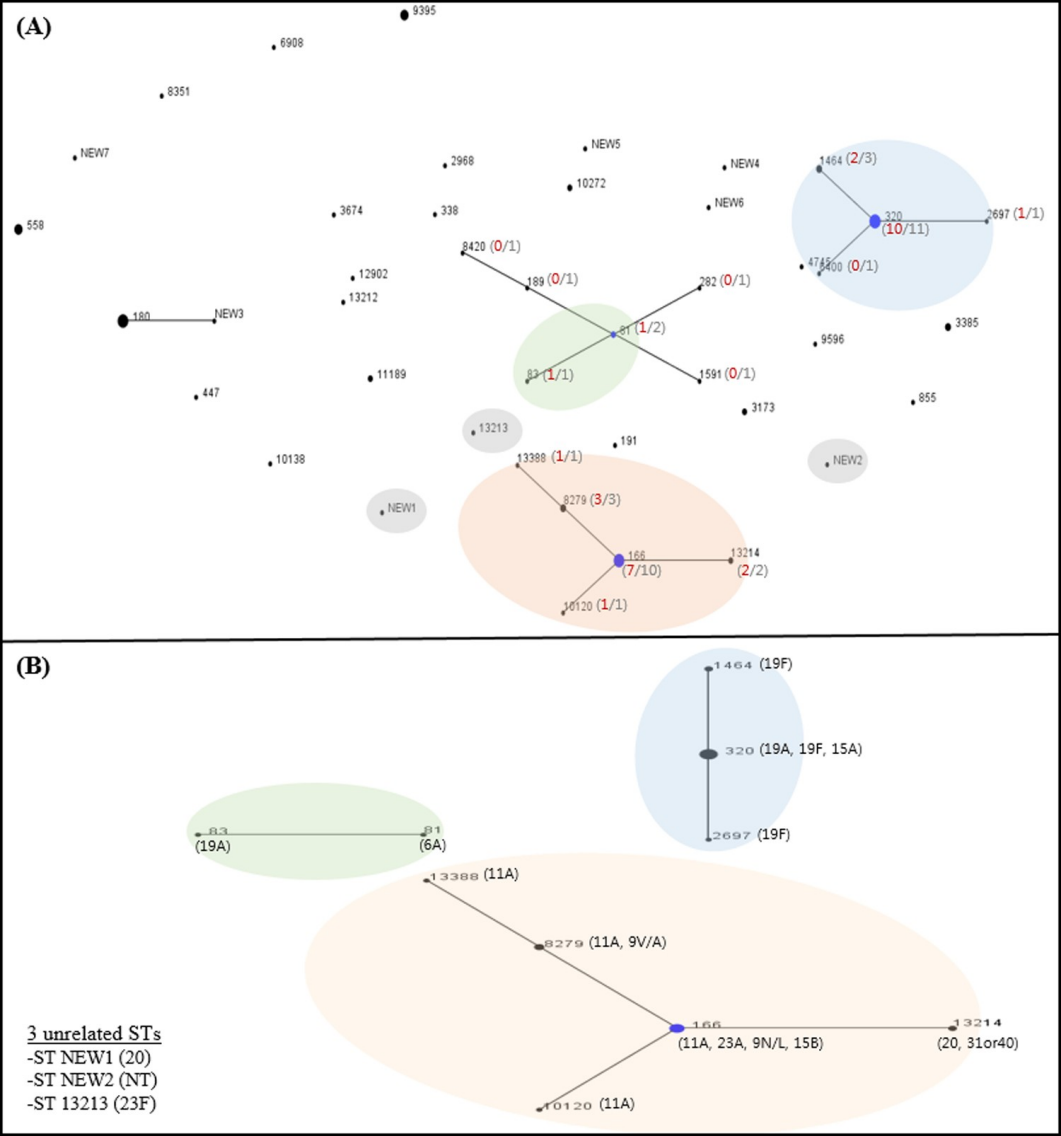
(A) eBURST of all 92 isolates. Colored circle indicates ceftriaxone non-susceptible isolates. (B) eBURST of 32 ceftriaxone non-susceptible isolates.

The relationships among all isolates in the MLST database are shown in Fig 1. Among 92 isolates, a total of 43 STs were identified. Seven allelic profiles (new 1 to new 7) were not recognized within the available MLST database of *S*. *pneumoniae*. eBURST analysis revealed that all 92 isolates were categorized into four major CCs (17 STs with 51 isolates) and 26 singletons (41 isolates), and each CC included two to six STs that differed at one or two alleles in the seven loci (Fig 1A). The allelic profiles and serotypes of CNSP are shown in Table 5 and Fig 1B. Thirty-two CNSP were categorized into three major CCs (10 STs with 29 isolates) and three singletons (3 isolates). CC320 included three STs (ST320, ST2697 and ST1464) combined with three serotypes. CC166 included five STs (ST166, ST10120, ST13214, ST8279, and ST13388) associated with seven serotypes. CC81 included only two isolates, which were ST81 and ST83, respectively. Although the first two large clusters of the three above mentioned included isolates with various serotypes, one particular serotype dominated each cluster (19A in CC320, and 11A in CC166, respectively). Overall, ST320 (10 cases), ST166 (7 cases), and ST8279 (3 cases) were dominant among 32 CNSP, and ST8279 was only detected in prior long-term care facility residents. The other two isolates found among long-term care facility residents were also closely related to ST8279: one isolate differed only at *ddl* (profile 251-11-10-1-6-1-441), and the other was a three-locus variant (profile 15-11-10-1-315-1-113). Two ST13214 (serotypes 20 and 31/40), three ST8279 (two serotype 11A and one 9V/A), one ST13388 (serotype 11A) and one new sequence type (serotypes 20) isolates were resistant to both ceftriaxone and levofloxacin.

**Table 5:** Distribution of sequence types and serotypes among 32 ceftriaxone non-susceptible *S*. *pneumoniae* isolates.

Clonal complex	Sequence type (ST)	No. of isolates (%)	No. of MDR	LTCF residents (No.)	Serotypes (No.)
320	320 (4-16-19-15-6-20-1)	10 (31.3%)	10	No	19A(8), 19F(1), 15A(1)
	2697 (4-16-19-15-6-20-252)	1 (3.1%)	1	No	19F(1)
	1464 (4-16-19-15-6-20-106)	2 (6.3%)	2	No	19F(2)
166	166 (7-11-10-1-6-1-1)	7 (21.9%)	7	No	11A(3), 23A(2), 9N/L(1), 15B(1)
	10120 (7-11-10-1-6-1-106)	1 (3.1%)	1	No	11A(1)
	13214 (15-11-10-1-6-1-1)	2 (6.3%)	2	No	20(1), 31 or 40 (1)
	8279 (251-11-10-1-6-1-1)	3 (9.4%)	3	Yes (3/3)	11A(2), 9V/9A(1)
	13388 (251-11-10-1-6-1-441)	1 (3.1%)	1	Yes (1/1)	11A(1)
81	81 (4-4-2-4-4-1-1)	1 (3.1%)	1	No	6A(1)
	83 (4-4-2-4-6-1-1)	1 (3.1%)	1	No	19A(1)
Others	13213 (15-29-4-16-30-1-26)	1 (3.1%)	1	No	23F(1)
	New 1 (15-11-10-1-315-1-113)	1 (3.1%)	1	Yes (1/1)	20(1)
	New 2 (425-275-118-135-292-595-178)	1 (3.1%)	1	No	NT(1)
Total, No. (%)		32 (100%)	32 (100%)	5 (15.6%)	

MDR, multidrug resistance; LTCF, long-term care facility; NT, non-typeable

## Discussion

*S*. *pneumoniae* is the most common causative pathogen of community-acquired pneumonia (CAP) and healthcare-associated pneumonia. Even in the era of PCV13, *S*. *pneumoniae* still accounts for 10–15% of CAP in developed countries [3, 23]. Thus, as in the case of hospitalized patients with CAP, either a beta-lactam plus a macrolide or a quinolone alone is recommended based on the guidelines of the Infectious Diseases Society of America and the American Thoracic Society [24]. However, the prevalence of antibiotic resistance has increased worldwide, particularly among adults [7, 8]. In Asian countries, more than 10% of pneumococcal isolates were resistant to ceftriaxone and levofloxacin [9, 25]; 38% of non-meningeal invasive isolates were resistant to ceftriaxone in Taiwan [26], while 14.1% of respiratory isolates were non-susceptible to levofloxacin in South Korea [25]. Consistent with these reports, pneumococcal isolates showed high resistance rates to ceftriaxone (non-susceptible in 34.8%, 32/92; resistant in 17.4%, 16/92) and levofloxacin (resistant in 13.0%, 12/92) in this study. Moreover, eight cases (8.7%) were resistant to both ceftriaxone and levofloxacin at the same time. Such a high antibiotic resistance might lead to inappropriate empirical antibiotic therapy, resulting in poor clinical outcomes. In this study, we evaluated the risk factors and clinical significance of CNSP acquisition, and analyzed the serotype and genotypic characteristics of these resistant isolates.

Identifying the risk factors of CNSP acquisition is important to provide effective antibiotic treatment. Here, chronic lung and renal diseases were independent risk factors for acquisition of CNSP. Patients with end-stage renal disease and chronic lung disease are at increased risks of pneumococcal disease. Although data on carriage are lacking, these patients with chronic lung disease and chronic renal failure would have a high density of pneumococci in the nasopharynx and may have acquired MDR pneumococci with repeated exposure to antibiotics [27, 28]. In patients with end-stage renal disease, monocytes, neutrophils, and dendritic cells are functionally impaired. As reported in experimental mouse studies, dysfunctional macrophages and impaired monocyte recruitment might result in decreased pneumococcal clearance in the nasopharynx [27]. In patients with chronic lung diseases, receptors for pneumococci, such as platelet-activating factor receptor, are overexpressed in the respiratory tract, increasing the risk of chronic colonization and invasive infections [29]. Besides these chronically ill patients, long-term care facility residents had high risks for CNSP infections. Because residents of long-term care facilities had several underlying medical conditions (diabetes mellitus and cerebrovascular disease), they were more likely to be heavily colonized with pneumococci and might be exposed to repeated antibiotic use. Thus, these individuals were more likely to be colonized with antibiotic-resistant pneumococci. In sequence, pneumococci would be transmitted to other patients by caregivers who were not strictly educated on infection control and cared for multiple patients at once. Several outbreaks of MDR pneumococcal pneumonia among long-term facilities have been reported [30, 31]. Here, all five CNSP isolates from long-term care facility residents were resistant to both ceftriaxone and levofloxacin. The population aged >65 years in South Korea has been continually increasing, reaching 13.5% in 2016, and >1,500 long-term care facilities are currently operating nationwide [32, 33]. Since long-term care facilities are an area of concern as potential sources of the spread of MDR pneumococci, active surveillance and interventions are required.

As antimicrobial resistance increases in pneumococci, therapeutic options become limited, and concerns regarding clinical outcomes may arise. The clinical relevance of the in vitro susceptibility of *S*. *pneumoniae* isolates has been controversial. Some studies have reported that antibiotic resistance in *S*. *pneumoniae* is not clinically relevant [34–36], whereas others have suggested higher mortality rate among patients infected with antibiotic-resistant *S*. *pneumoniae* [37–39]. In addition, data are insufficient on the clinical impact of ceftriaxone resistance, because most studies focused mainly on penicillin resistance [34, 37]. Moreover, most studies focusing on ceftriaxone resistance did not use the revised CLSI breakpoints of ceftriaxone for non-meningitis isolates [35, 36]. In this study, ceftriaxone or other antibiotic resistance were not associated with poor clinical response. It is unclear whether pneumococcal infections with low-level ceftriaxone resistance (MIC 4 μg/mL) would respond to cephalosporin therapy or not. When comparing pneumococcal infections with high-level resistance to ceftriaxone (MIC ≥8 μg/mL) to the CSP group, the 5-day response tended to be better in the CSP group, although this finding was statistically insignificant (33.3% vs. 69.8%, *p* = 0.164) (data not shown in the results). The emergence of high-level resistance to cephalosporin is an alarming issue, and further studies are required regarding clinical outcomes, focusing on ceftriaxone resistance. Considering the high drug resistance profile and poor clinical outcomes among residents of long-term care facilities, pneumococcal vaccination in these individuals should be emphasized.

Regarding the serotype distribution, this study revealed significant changes in the distribution of serotypes after introducing PCV13. The PCV13 serotype coverage rate (42.4%) in this study was lower compared to that during 2002–2005 (65.7%)[40]. However, vaccine serotypes, especially the serotypes included only in PPSV23, were significantly higher in the CNSP group. The most common serotypes in the CNSP group were serotypes 19A, 11A, and 19F, which were known to be highly drug resistant [18, 26, 40–42]. Serotype 19A, which was a significant cause of morbidity and mortality before the introduction of PCV13 in 2010 [43, 44] was an MDR serotype [26, 41], and MDR ST320 was as a major genotype of serotype 19A [41, 45]. This study shows that serotype 19A/ST320 pneumococcus are still prevalent in Korea in the era of PCV13, under the influence of unrecognized factors besides vaccine selection pressure. The high level of antimicrobial resistance might have an effect on the increase in serotype 19A pneumococci [46]. In the US, there was a remarkable shift in the serotype 19A genetic structure, where the prevalence of highly resistant serotype 19A strains (CC320) continued to increase with a concurrent decrease in the prevalence of CC199 [46, 47]. In agreement with previous reports [18, 48], serotypes 11A and 19F were common serotypes in the present study, especially in the CNSP group. Serotype 19F is known to have a higher MDR rate [26, 40, 48], and an association with ST320 was observed in this study. The increase in serotype 11A prevalence may result partly from PCV introduction. The prevalence of serotype 11A in Korean hospitals was only 3.8% in 1996–2001, the period before the introduction of PCV [49]. This proportion increased to 8.0–13.2% in 2011–2013 [18], a rate similar to the findings of our study. The increase in serotype 11A isolates was also thought to be attributable to the clonal expansion of CC166 isolates, which showed very high antimicrobial resistance rates, because antimicrobial resistance has been considered to be one of the key factors influencing the survival of bacterial clones [18]. Levofloxacin resistance was also identified among serotype 11A isolates in previous Korean reports [50], and non-susceptibility to levofloxacin was associated with healthcare-associated factors and cerebrovascular disease [25]. ST 8279, a CC166 isolate known to be MDR, and even an XDR clone [42, 50], was previously reported among long-term care facility residents who underwent tracheostomy [42]. Similar findings were observed in this study: three isolates among five CNSP with long-term care facility residents were ST8279, the other two isolates were also closely related to ST 8279, and all of these were XDR. It is possible that XDR ST8279 is spreading in long-term care facilities, and should be monitored closely. Pneumococcal vaccines covered the majority of the serotypes that conferred a CNSP phenotype (PCV13, 50%; PPSV23, 81.2%), emphasizing the importance of vaccination for at-risk individuals with comorbid conditions. Continuous monitoring of the dynamic nature of both ceftriaxone resistance and serotype distribution in *S*. *pneumoniae* is crucial to understanding the impact of pneumococcal vaccination in both children and adults.

In conclusion, a distinct group of patients with chronic lung/renal diseases and residents of long-term care facilities appears to be an important reservoir of CNSP in South Korea. These CNSP isolates might have emerged both de novo after repeated exposure to antibiotics and by means of person-to-person transmission in hospitals or long-term care facilities. We revealed that 81.2% of the ceftriaxone non-susceptible isolates belonged to PPSV 23 serotypes, and serotype 19A, 11A, and 19F pneumococci were predominant. Specifically, serotype 11A was associated with XDR ST8279, which is closely associated with long-term care facility residents. Our findings call for attention and collaborative strategies to monitor the spread of these strains among long-term care facilities.

## Supporting information

S1 File Demographic, clinical and microbiological data of pneumococcal isolates used in this study. (XLSX)
